# Pulsed Radiofrequency and Platelet Rich Plasma in Degenerative Joint Arthritis: Two Case Reports and Literature Analyses

**DOI:** 10.3390/life13061334

**Published:** 2023-06-07

**Authors:** Luca Gregorio Giaccari, Francesco Coppolino, Caterina Aurilio, Maria Caterina Pace, Maria Beatrice Passavanti, Vincenzo Pota, Dario Alicino, Giuseppe Pulito, Pasquale Sansone

**Affiliations:** 1 Department of Anesthesia and Intensive Care, “Vito Fazzi” Hospital, 73100 Lecce, Italy; 2 Department of Women, Child, General and Specialistic Surgery, University of Campania “L. Vanvitelli”, 80138 Naples, Italy

**Keywords:** osteoarthritis, Platelet Rich Plasma (PRP), Pulsed radiofrequency (PRF)

## Abstract

(1) Background: Osteoarthritis (OA) is a debilitating joint disease. The are several therapies available for OA. According to current knowledge, the combination of Platelet-Rich Plasma (PRP) and Pulsed Radiofrequency (PRF) can be applied in the treatment of pain of nociceptive origin due to peripheral tissue damage. (2) Methods: We performed a narrative review identifying the articles by searching electronic databases. A retrospective analysis of patients with OA treated with PRF and PRP in “Vito Fazzi” Hospital (Lecce, Italy) was performed. (3) Results: A total of four publications on the use of PRP and PRF in degenerative joint arthritis were included in our review. In our experience, two patients with OA were treated with PRP and PRF after unsuccessful conservative treatment. Patient pain score, daily activity ability, active range of activity, and muscle strength improved after treatment. Patients reported a higher level of satisfaction. No major adverse events were reported. (4) Conclusions: The goal of the combined application of the two treatments is to make full use of the analgesic effect of PRF and the repairing effect of PRP. At present, the therapeutic potential of PRP and PRF in OA remains unmet.

## 1. Introduction

Osteoarthritis (OA) is a debilitating joint disease that causes the destruction of the articular cartilage and underlying bone; the etiopathogenesis is multifactorial and includes the gradual disappearance of articular cartilage, osteophyte formation, subchondral bone change, and aseptic synovitis [1].

Worldwide, OA is the most common joint disorder. Approximately 85% of persons more than 75 years of age have symptomatic OA; 40% of them have significant difficulties in daily activities interfering with work or social activities. OA is characterized by joint pain, which worsens with activity, especially after a period of rest, the so-called “gelation phenomenon” [1]. The joints most affected are the hands, knees, hips, and spine, usually asymmetrically [1]. Diagnosis is primarily clinical, based on the patient’s history and clinical examination. Radiography is helpful in confirming the diagnosis, while more advanced imaging techniques (computed tomography or magnetic resonance imaging) are rarely helpful [1]. The therapies available for OA can be classified into three groups: pharmacological (acetaminophen, nonsteroidal anti-inflammatory drugs, opioids, and duloxetine), non-pharmacological/non-surgical (e.g., physiotherapy, balance exercises, and rehabilitation), and surgical therapies (e.g., total joint replacement) [1]. The guidelines present a stepwise approach to the treatment of OA.

Platelet-Rich Plasma (PRP) is defined as a volume of plasma with a higher-than-normal concentration of platelets. This concentration is at least 1,000,000 platelets/mm^3^, usually five times higher than normal [2]. Traditionally, PRP is produced after the centrifugation of autologous blood [3]. Once PRP is obtained, platelets can be activated by various substances such as calcium chloride or thrombin [3]. PRP was first used in open heart surgery in 1987. Since the 1990s, it has been used in maxillofacial surgery, dentistry, and vascular surgery. Thanks to its excellent properties, PRP began to be used in orthopedics and sports medicine. Recent application fields include cosmetic and plastic surgery [4]. Patients undergoing treatment for OA can experience improved clinical outcomes with PRP [5,6]. PRP contains several growth factors, such as transforming growth factor beta (TGF-β), platelet-derived growth factor (PDGF), vascular endothelial growth factor (VEGF), and other cytokines, such as insulin-like growth factor1 (IGF-1) or hepatocyte growth factor (HGF), that can stimulate healing of soft tissue and joints [3].

The use of radiofrequency (RF), conventional or pulsed, is a technique commonly used for the treatment of chronic pain in various musculoskeletal disorders, such as low back pain [7]. Pulsed radiofrequency (PRF) has been used in the treatment of pain for more than 30 years. PRF was invented and developed by Menno E. Sluijter and applied to humans for the first time in February 1996 [8,9]. The clinical effects of PRF derive from several mechanisms due to the generation of a strong electromagnetic field around the tip of the electrode. This leads to the generation of heat and an electric field which induces changes in neuronal cells.

According to current knowledge, the combination of PRF and PRP can be used in the treatment of nociceptive pain due to peripheral tissue damage [10]. Michno et al. showed that the activation of blood platelets in PRP by PRF potentiated their secretion of alpha and dense granules in in vitro studies [11]. PRF does not affect platelet integrity, metabolism, and function [12]. Other in vitro studies have proven low-frequency pulsed electromagnetic fields (PEMF) can improve bone, cartilage, and tendon tissue healing [13,14,15]. Furthermore, a synergistic effect was observed when human tenocytes were exposed to electrical stimulation after the administration of PRP [14]. A combination of both PRF and PRP applications may be useful in controlling pain and accelerating healing in regenerative medicine [16]. Since, to our knowledge, only a few randomized controlled trials (RCTs) have been published investigating the combined use of PRP and PRF in the treatment of OA, evaluation of their efficacy is based, in part, on case reports and observational studies. In the absence of rigorous cohort studies, the best available evidence may be provided by these studies describing efficacy in individuals with OA receiving PRP and PRF, published in the peer-reviewed literature. While it is clear that RCTs are the appropriate methodology for determining efficacy and safety, case studies are often used to gain insight into the use of new techniques. In the case of OA, case reports of efficacy and safety events associated with PRP and PRF should best serve as a basis for conducting RCTs.

Aims. The aim of our article is to focus on the available relevant evidence that supports the use of PRP and PRF in osteoarthritis, firstly performing a narrative review and then reporting a case series of patients with OA treated with PRP and PRF after unsuccessful conservative treatment in our hospital.

## 2. Materials and Methods

We performed a narrative review identifying the articles by searching electronic databases (PubMed, Embase, Scopus, Google Scholar, and Cochrane Library). Other relevant publications were identified from the bibliography. The search terms used were “Osteoarthritis”, “Platelet-Rich Plasma”, and “Radiofrequency Therapy”. The titles and abstracts were screened by two researchers (L.G.G. and F.C.) to identify the keywords. The selected papers were read in full by two of the authors independently, and, in case of disagreement, a third reviewer (P.S.) was consulted. The first search was conducted on 1 February 2023. All publications up to the end of January 2023 were included in this review. This review is based on previously conducted studies. We applied no language restrictions in searches.

The inclusion criteria were as follows: (1) the full study was published; (2) the study described clinical use of PRF and PRP in degenerative joint arthritis; (3) the study reported the clinical outcome of the patient(s) treated with PRF and PRP. Exclusions criteria were as follows: (1) the study did not report clinical outcome; (2) the study had duplicate data with others (in these cases, only the largest study was retained); (3) the study presented pooled data that did not allow for extrapolation of useful information.

In addition to evaluating study characteristics, we analyzed the visual analog scale (VAS) at various time points, parameters to measure articular motility, and any adverse reactions. Possible adverse effects include PRP incompatibility reactions, surgery site swelling, tissue necrosis, bleeding, local inflammation, infection, or local pain.

The data were analyzed using standard computer programs (Excel, Microsoft Office 2021). Results are reported as mean ± standard deviation (SD). Data consistency was assessed using the Chi-squared test with a 95% confidence level. Comparison was performed using Student’s *t*-test with a statistical significance level of *p* < 0.05.

A retrospective analysis of patients with OA treated with PRF and PRP in the Pain Clinic of the “Vito Fazzi” Hospital (I° floor—Padiglione Oncologico “Giovanni Paolo II”—Lecce, Italy) was performed since January 2020.

## 3. Results

A total of four publications on the use of PRP and PRF in degenerative joint arthritis were included in our review. Three case reports [17,18,19] and one randomized controlled trial (RCT) [20] published between 2018 and 2021 were included. The studies’ characteristics are reported in Table 1.

In the studies included, 39 patients (15 males and 24 females) were treated first with PRF and thereafter with autologous PRP injection in the same session. The mean age was 55.73 ± 3.82 years. Thirty-four patients were diagnosed with knee osteoarthritis. Four patients reported supraspinatus injury. Avascular necrosis of the femoral head was diagnosed in a case.

In all studies, PRF was carried out at 42 °C for 120–240 s. PRP was prepared according to the local protocol. After local anesthetic infiltration, 4–20 mL of autologous PRP was injected.

Patients were followed up at 1, 3, and 6 months post-procedure (see Table 2). Pre-procedure pain was 7.79 ± 1.12. The pain was 2.83 ± 0.59 at 1 month, 2.07 ± 0.44 at 3 months, and 1.52 ± 0.52 at 6 months.

A comprehensive evaluation of the patient’s pain, daily activity ability, active range of activity, and muscle strength improved 6 months after treatment. No adverse events were reported, such as wound swelling, exudation, fever, nerve injury, and vascular injury. No antibiotics were used.

We report two cases (1 male and 1 female) treated at the Pain Clinic of the “Vito Fazzi” Hospital in Lecce (Italy). The patients were informed prior to the publication of this article, and they provided written informed consent to data use.

Case 1. A 67-year-old patient presented with severe pain (VAS score 8/10) in the left knee. About 10 years ago, they reported a fracture of the right lateral tibial condyle; several traumas to the left knee were reported, but no documentation was available. For some years, they suffered from minor pains that increased in recent months. No history of surgery or any other treatment was reported. On examination, a limping gait was observed. Tenderness around the left knee joint, moderate swelling, increased pain on weight-bearing, and decreased ROM were observed. The X-ray of the left knee was grossly normal. They underwent an MRI of the bilateral knee joint with evidence of marked tricompartmental gonarthrosis with the osteophytotic reaction on the left. No history of steroid use was reported. The patient reported the use of nonsteroidal anti-inflammatory drugs, tramadol, vitamin D, and calcium supplements.

Case 2. A 76-year-old patient presented with severe pain (visual analog scale [VAS] score 7/10) in the right knee. No past traumas to the right knee were reported. The patient reported the onset of mild pain for some years, which had increased in recent months. On examination, a limping gait was observed. Tenderness around the right knee joint, moderate swelling, increased pain on weight-bearing, and decreased ROM were observed. X-ray of the right knee showed marked tricompartmental gonarthrosis with slightly greater changes in the medial femorotibial compartment. No history of steroid use was reported. The patient reported the use of nonsteroidal anti-inflammatory drugs, vitamin D, and calcium supplements.

Both patients were treated first with PRF and then with an injection of autologous PRP in the same session. The combined use of PRP and PRF was proposed when the following criteria were met:

– Pain lasting more than 3–6 months with VAS > 4;
– Clinical data, images, and diagnostic procedures confirming the presence of OA;
– Symptoms resistant to conservative therapies such as NSAIDs and physiotherapy;
– The patient’s goal was to avoid surgery.

Informed consent was obtained by providing information on risks (e.g., infection, bleeding, and tissue injury), benefits, and possible alternative therapies.

PRP and PRF were contraindicated in the presence of the following:

– Active infections;
– Use anticoagulants and antiplatelet agents;
– Blood dyscrasias;
– Use systemic immunosuppressive drugs.

All procedures were performed after local anesthesia was obtained in the operating room. After palpating the anterior patella region with the knee angled at 30–45 degrees, the patella was visualized with the aid of a 5–10 MHz linear probe. The transducer was then moved proximally until the patella disappeared. After acquiring the best ultrasound image at this location, the skin was marked according to the location of the probe. After disinfection, local anesthesia was performed using 1% lidocaine. Then, a 22-gauge Quincke needle was inserted into the knee joint under ultrasound guidance. A total of 2 mL of 1% lidocaine was injected into the joint while observing the filling of the suprapatellar bursa in real time. Once proper placement was confirmed, the stylet was withdrawn, and the RF probe was inserted through the introducer needle. PRF was administered for 15 minutes at 42 °C and a pulse span of 20 ms at 2 Hz.

After drawing 60–120 mL of blood from the patient into tubes containing an anticoagulant, we used the Z206A Compact Centrifuge (HERMLE, Wehingen, Germany). Our protocol was based on double centrifugation at 1000 rpm for 10 min, followed by 4000 rpm for 10 min. This protocol produced a five-fold increase in platelet concentration above the baseline level and preserved platelet integrity. Then, approximately 20 mL of intra-articular PRP was injected using another entry site under ultrasound guidance.

Patients had to rest for 48 h and inform the physician of any joint abnormalities, especially signs of inflammation. They were advised of the possibility of more pain during the first few days after the procedure and encouraged to practice physical therapy before and after treatment to improve their prognosis.

**Figure 1:**
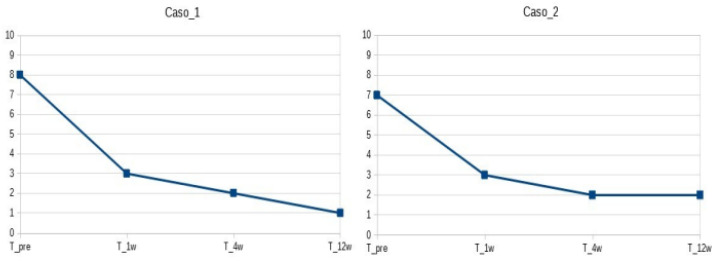
Pain evaluation (VAS) at baseline and after treatment (1, 4, 12 weeks).

Patients were followed up 1, 4, and 12 weeks after the procedure (see Figure 1). At baseline, VAS was 8 and 7, respectively. After 1 week, the pain was 3/10. After 4 weeks, patients had VAS 2/10 and were able to perform all of their activities with minimal discomfort. After 12 weeks, patients had VAS 1-2/10 (occasional pain when climbing stairs). Both patients could walk independently without aid. At the last follow-up visit, knee ROM values were −10 degrees of knee extension and 100 degrees of knee flexion for the first patient, −5 degrees of knee extension, and 120 degrees of knee flexion for the second patient.

No adverse events were reported.

## 4. Discussion

OA is a serious chronic disease that affects one in seven adults in the United States, about 32.5 million people. OA causes pain, stiffness, and swelling, which cause limitations that interfere with the ability to perform routine daily living activities. In addition to traditional treatments, others are being developed, especially in the field of regenerative medicine. There are several clinical studies that showed good results in treating joints arthritis with PRP or PRF. Very few reports in the literature in which PRF and PRP combination are reported. The clinical studies published to date are publications with a limited number of patients.

In 2018, Kothari et al. treated a 53-year-old patient with avascular necrosis of the femoral head with PRF of articular branches of the femoral and obturator nerve and intraarticular injection of PRP [17]. After 12 weeks, the combination therapy resulted in a significant improvement in VAS pain score, function, and ROM.

Sixty patients with chronic OA of the knee were enrolled in a randomized study by El-Tamboly et al. [20]. The authors compared genicular nerve ablation with RFP and the combined use of intra-articular radiofrequency and PRP. The patients of the second group showed a significant improvement in pain score and joint function parameters compared to the PRF of the genicular nerve.

In 2021, Jin et al. combined PRF with four injections of PRP into the knee joint to treat OA [18]. Pain reduction and recovery of knee joint mobility were observed. The goal of the combined application of PRF and PRP was to benefit from the analgesic effect of PRF in combination with the reparative effect of PRP. In conclusion, it was verified that the combined application of PRF and PRP could improve knee osteoarthritis symptoms and shorten the duration of treatment.

The combination of PRF and PRP has also been used for the treatment of supraspinatus lesions by the same authors. Jin et al. treated four patients with a single injection of PRP after the PRF procedure [19]. Patients reported improvement in both pain perception and joint movement. All these results were demonstrated at 6 months.

In our experience, we used PRP and PRF as both work via different mechanisms, and thereby, we have a synergistic effect of regeneration and pain relief. Two patients with OA were treated with PRP and PRF after unsuccessful conservative treatment. Patients refused surgery for various reasons, particularly the need to return to normal activity quickly and the desire not to undergo surgery. VAS pain score, daily activity ability, active range of activity, and muscle strength improved 12 weeks after treatment. Patients reported a higher level of satisfaction. No major AEs were reported.

Management of OA may include educational, behavioral, psychosocial, and physical interventions, as well as topical, oral, and intra-articular medications. Therapeutic decisions must be made considering the personal beliefs and preferences of the patient, as well as his medical condition. In the current guidelines for the management of osteoarthritis [21], PRP treatment is strongly recommended in patients with knee and/or hip OA. This is mainly due to the lack of standardization in the available preparations of PRP as well as in the techniques used. Radiofrequency ablation is conditionally recommended for patients with knee OA. This is due to the heterogeneity of the ablation techniques and the lack of long-term safety data.

Despite the many options available, some patients continue to be symptomatic or experience negative effects from available interventions.

Recent studies suggest that PRP could be a promising therapy for OA [22,23]. Currently, the PRP preparation method and injection protocol are not standardized. Published studies suggest that double centrifugation (1000–1600 rpm followed by 2500–4000 rpm) for 10–15 min yields the greatest increase in platelet concentrations [22,23]. Intra-articular PRP injection therapy seems to be a safe treatment with the potential to provide symptomatic benefits for OA. Systematic reviews have identified LP-PRP as the optimal PRP formulation to achieve the best results in the treatment of OA [24]. Activated platelets release several protein growths and angiogenic factors involved in the healing process. The levels of platelet growth factors such as PDGF and TGF-β in PRP had no influence on cell activity. Conversely, the increase in IGF-1 levels, which do not depend on platelet concentration, is directly correlated with increased cell proliferation [25]. In in vitro conditions, exogenous activators such as thrombin, collagen, or CaCl2 are used [26,27]. After injection, PRP is exposed to collagen in the tissue, and blood platelets are activated partially and slowly [27]. Frelinger et al. demonstrated that stimulation obtained with a pulsed electric field (PEF) could provide more consistent platelet activation and avoid the complications associated with the use of bovine thrombin [28]. Subsequently, they demonstrated in in vitro samples that the application of low electric field pulses results in a lower generation of platelet microparticles but with overall activation levels similar to those obtained with thrombin [29]. Recently, Michno et al. investigated whether PRF may potentiate the activation of platelets in PRP samples when both these techniques are combined together in in vitro conditions [11]. They concluded that a combination of both PRF and PRP methods might provide a more effective opportunity for tissue regeneration in several clinical fields. Other in vitro studies have confirmed the role of PRF as an activator [12,13,14].

All these data indicate that a combination of both PRF and PRP applications may be useful in controlling pain and accelerating healing in OA.

Limitations. First, our review includes a few studies (3 case reports and 1 RCT), and most of these involve a few patients. Due to the greater potential for bias, case series and observational studies are often excluded from reviews. In a typical review of developing technology, that is, the combination of PRF and PRP for the treatment of OA, case series, and observational studies contribute substantially to the available evidence, and their results supplement the limited evidence available from other studies. Second, the preparation method of PRP in these studies is not standardized. The different existing protocols for the preparation of PRP are one of the reasons that can explain the variability of the results obtained in the different studies conducted. Third, the PRP and PRF therapy protocol is not uniform in the analyzed studies. Finally, we reported only two cases treated with PRP and PRF therapy in our pain clinic. In Italy, treatment with PRP is still reserved for only a few extremely selected cases.

## 5. Conclusions

In this work, we have reviewed currently available evidence supporting the use of PRP and PRF to treat OA. The goal of the combined application of the two treatments is to make full use of the analgesic effect of PRF and the repairing effect of PRP in order to maximize the advantages of these treatment methods. This approach could be promising in unresponsive patients to conservative therapies and where surgical treatment is contraindicated.

In conclusion, at present, the therapeutic potential of PRP and PRF in OA remains unmet, and in the absence of standardization, its clinical efficacy will still remain an open debate. Randomized trials with larger numbers of patients need to be conducted.

## Figures and Tables

**Table 1:** Studies characteristics.

Author, Year	Study (*n*)	Disease	Outcomes
Kothari et al., 2018 [17]	Case Report (1)	Avascular necrosis femoral head	VAS, ROM, Harris Hip scores
El-Tamboly et al., 2021 [20]	RCT (60)	Knee osteoarthritis	VAS, WOMAC, IKDC, knee US
Jin et al., 2021 [18]	Case Report (4)	Supraspinatus injury	VAS, Constant–Murley shoulder score, daily activity ability, ROM
Jin et al., 2021 [19]	Case Report (4)	Knee osteoarthritis	VAS, WOMAC

IKDC, International Knee Documentation Committee; RCT, Randomized Controlled Trial; ROM, Range Of Motion; WOMAC, Western Ontario and McMaster Universities Arthritis Index; US, UltraSound; VAS, Visual Analogue Scale.

**Table 2:** Pain evaluation (VAS) at baseline and after treatment (1 week and 1, 3, 6 months).

	VAS				
	T_0	T_1w	T_1m	T_3m	T_6m
Kothari et al., 2018 [17]	8	3	2	1–2	–
El-Tamboly et al., 2021 [20]	5.9	–	–	1.8	0.80
Jin et al., 2021 [18]	8.75	–	3.25	2.5	1.75
Jin et al., 2021 [19]	8.5	–	3.25	2.5	2.0
Total (±SD)	7.79 ± 1.12	3	2.83 ± 0.59	2.07 ± 0.44	1.52 ± 0.52

## Data Availability

Data is available upon reasonable request.
